# Ultrasound Evaluation of the Cervix to Predict Failed Labor Induction

**DOI:** 10.1055/s-0039-1693679

**Published:** 2019-08

**Authors:** Christian Eric Sevrin, Letícia Matheuz Martorelli, Eduardo Augusto Brosco Famá, César Eduardo Fernandes, Mauro Sancovski, Emerson Oliveira

**Affiliations:** 1 Department of Obstetrics and Gynecology, Faculdade de Medicina do ABC, Santo André, SP, Brazil

**Keywords:** cervix uteri, endosonography, obstetric delivery, induced labor, prolonged pregnancy, colo do útero, endossonografia, parto obstétrico, trabalho de parto induzido, gravidez prolongada

## Abstract

**Objective** Labor induction does not always result in vaginal delivery, and can expose both the mother and the fetus to the risks inherent to the induction procedure or a possible cesarean section. Transvaginal sonography (TVS) of the cervix is a useful tool to predict prematurity; in the present study, this tool was used to evaluate postterm induction.

**Methods** We evaluated the ultrasound characteristics of the cervix (cervical length, cervical funneling, internal os dilation, the presence or absence of the cervical gland area [CGA], and the morphological changes of the cervix as a result of applying fundal pressure) before the onset of labor induction among women with postterm pregnancy to identify the possible predictors of failed labor induction. The Bishop score (BS) was used for comparison purposes. Three groups were evaluated: successful versus unsuccessful induction; vaginal delivery versus cesarean delivery (excluding cases of acute fetal distress [AFD]); and vaginal delivery versus cesarean delivery (including cases of AFD). A fourth group including only the primiparous women from the three previous groups was also evaluated.

**Results** Based on the studied characteristics and combinations of variables, a cervical length ≥ 3.0 cm and a BS ≤ 2 were the best predictors of induction failure.

**Conclusion** Although TVS is useful for screening for induction failure, this tool should not be used as an indication for cesarean section.

## Abstract (pt)

**Resumo:**

**Objetivo** Nem sempre a indução do parto termina em parto vaginal, expondo tanto a mãe quanto o feto aos riscos inerentes ao procedimento de indução, ou a uma possível cesárea. A ultrassonografia transvaginal (UTV) se mostrou interessante instrumento na predição da prematuridade e, neste estudo, utilizamos este instrumento na situação inversa: indução do parto no pós-datismo.

**Métodos** Avaliamos variáveis ultrassonográficas do colo uterino (comprimento, presença de afunilamento, dilatação do orifício interno do colo, eco glandular endocervical [EGE] evidente ou não, e alterações morfológicas do colo uterino à compressão fúndica uterina) antes do início da indução em gestantes com pós-datismo, na tentativa de encontrar um possível preditor de falha de indução. O índice de Bishop (IB) também foi utilizado para fins de comparação. Três grupos foram avaliados: indução bem-sucedida x malsucedida; parto vaginal x cesárea (excluindo casos de sofrimento fetal agudo[SFA]); e parto vaginal x cesárea (incluindo casos de SFA). Além disso, um quarto grupo composto apenas pelas primíparas dos outros três grupos também foi avaliado.

**Resultados** Com base em todas as características estudadas e combinações de variáveis, o comprimento do colo uterino ≥ 3,0 cm e IB ≤ 2 foram os melhores preditores em todos os grupos analisados.

**Conclusão** Apesar de a UTV do colo uterino ser um bom exame para rastreamento de indução malsucedida, não deve ser usado para se indicar uma cesariana.

## Introduction

Despite significant medical advances over the past few decades, the mechanisms that trigger the onset of labor have not yet fully understood. Inflammatory activity mediated by gene expression is one mechanism that regulates this process.^1^ ^2^ ^3^ ^4^ ^5^ ^6^ Given the lack of understanding of the factors that trigger labor, we frequently manage situations involving increased pregnancy risks, including postterm births (> 40 weeks of gestation), postmature births (> 42 weeks of gestation), and the complications commonly associated with these conditions.^7^ ^8^

One strategy used to avoid postterm complications is to prevent the prolongation of pregnancy by initiating delivery. Two strategies can be used for this purpose: the induction of vaginal delivery or cesarean delivery. The incidence of cesarean births is high in Brazil, representing 50% of all births.^9^ ^10^ ^11^ Cesarean birth considerably increases the risks for the mother while decreasing the morbidity for the fetus.^12^ ^13^ The induction of vaginal delivery increases the risks for both the mother and the fetus; however, these risks are markedly reduced in cases in which the procedure is well-supervised.^14^ ^15^ ^16^

Another likely possibility is the failed induction of vaginal delivery, which leads to the need for cesarean section. In this case, the mother and the fetus are subject to the risks of both labor induction and cesarean section.^17^ ^18^ ^19^ Given the high risk of failure, recommending cesarean sections to women who are known in advance to be ineligible for labor induction might considerably reduce the risks involved in these pregnancies. Induction failure causes psychological trauma but precludes the benefits of natural birth.^20^

Transvaginal sonography (TVS) of the cervix is an important predictor of the risk of preterm birth, and its use has been widely studied.^21^ ^22^ ^23^ Like the prediction of prematurity, TVS has also been studied as a predictor of labor induction. Various studies have evaluated these variables. However, the sample sizes were small, and the methods for collecting and evaluating data were distinct, limiting comprehensive meta-analyses and systematic reviews.^24^ ^25^ ^26^ ^27^ We performed the present study to address these limitations. Furthermore, one objective of the present study was to evaluate the effectiveness of TVS to screen for labor induction in our population using our protocols.

## Methods

This prospective cohort study was conducted between 2016 and 2017 at Hospital da Mulher Maria José dos Santos Stein, which is affiliated with Faculdade de Medicina da Fundação do ABC (FMABC). The study was approved by the Ethics in Research Committee of our institution under protocol no. 1.193.101. A total of 95 pregnant women at gestational age (GA) > 40 weeks who were not in labor and had an intact amniotic sac were considered eligible for labor induction. Indications for labor induction included patients at 41 weeks of gestation or the presence of maternal and/or fetal comorbidities at > 40 weeks of gestation (Table 1).

**Table 1:** Demographic characteristics of the groups

		Analysis A			Analysis B			Analysis C			All Patients
Characteristics		Successful induction	Unsuccessful induction	*p*-value	Vaginal delivery	Caesarean delivery	*p*-value	Vaginal delivery	Caesarean delivery	*p*-value	
						(except for acute fetal distress)			(including acute fetal distress)		
*Number of patients*		69	11		62	18		62	28		90
*Race*	*Caucasian*	33	6	0.7529	28	11	0.289	28	14	0.819	42
	*Non-Caucasian*	36	5		34	7		34	14		48
*Parity*	*Primiparous*	40	11	0.0059	34	17	0.0018	34	27	< 0.0001	61
	*≥ Secondiparous*	29	0		28	1		28	1		29
*Maternal age (in years)*	*Mean (standard deviation)*	25 (6.1)	26 (6.1)	0.46	25 (6.3)	26 (5.4)	0.659	25 (6.3)	25 (5.4)	0.8	25 (6.3)
*GA (in days)*	*Mean (standard deviation)*	285 (3.3)	285 (2.7)	0.59	285 (3.4)	285 (2.6)	0.56	285 (3.4)	285 (3.9)	0.27	285 (3.5)
*Maternal body mass index*	*Mean (standard deviation)*	31 (5.7)	34 (4.0)	0.144	31 (5.7)	33 (4.7)	0.248	31 (5.7)	32 (4.8)	0.345	31 (5.5)
*Birthweigth (in grams)*	*Mean (standard deviation)*	3,408 (380.2)	3,384 (403.6)	0.846	3,418 (382.2)	3,358 (383.6)	0.555	3,418 (382.2)	3,428 (377.4)	0.912	3421 (378.6)
p: statistical significance											

The GA was calculated using the first day of the last menstrual period (LMP) in cases in which the difference in GA based on the initial obstetric sonography (OS) examination was within the OS margin of error, or the initial OS in cases in which the difference in GA was beyond the OS margin of error. In most cases, the initial OS was performed before 20 weeks of gestation; therefore, the GA showed adequate reliability.

Before inducing labor, the cervix was evaluated via TVS (Toshiba SSA-510A Diagnostic Ultrasound System, Minato, Tokyo, Japan) following the guidelines established by the Fetal Medicine Foundation (https://www.fetalmedicine.org). For this evaluation, only two physicians participated in the data collection to reduce interobserver variation. The TVS characteristics evaluated were the opposite of those used to evaluate the risk of prematurity, because part of the intention of the present study was to evaluate the difficulty of the evolution of birth.^21^ ^22^ ^23^ The ultrasound characteristics analyzed included cervical length (distance from the internal orifice to the external orifice), cervical funneling, internal os dilation, cervical gland area (CGA), the morphological changes of the cervix as a result of applying fundal pressure, and the absence of sludge.^28^ The absence of sludge was removed from the statistical analysis because none of the evaluated patients presented with this characteristic. After delivery, the preinduction TVS characteristics and the Bishop^29^ score (BS) were examined to determine whether they were associated with the method of childbirth.

The cervical length was defined as ≥ 0.5 cm to ≥ 4.5 cm with 0.5-cm intervals; the other TVS variables (cervical funneling, internal os dilation, CGA, and morphological cervical changes as a result of applying fundal pressure) were evaluated individually and in combination with a cervical length of ≥ 3.0 cm (this value was considered as the best cut-off point for the present as well as several other studies) for every possible combination.^25^ ^26^ ^27^ Similarly, the BS was evaluated serially from ≤ 5 (this score was considered unfavorable to labor induction, requiring the preliminary preparation of the cervix) to 1 on a 5-point scale.

The protocol for labor induction followed the guidelines established by the Department of Obstetrics at FMABC. The BS was calculated to guide the method to be applied. Membrane detachment was initially performed. Preinduction cervical ripening (BS ≤ 5) was performed by inserting a 25-mcg misoprostol tablet into the vagina every 6 hours (up to a maximum of 6 tablets), until the cervix reached a BS of ≥ 6. For labor induction (BS ≥ 6) and labor augmentation, 5 IU of oxytocin diluted in 500 mL of 5% glycosylated serum (GS) were used, starting at 2 mIU/min (4 drops/min) with increments of 2 mIU every 30 minutes until effective contractions were initiated; the maximum dose was 32 mIU/min (64 drops/min). Artificial membrane rupture can also be used as an alternative method for labor induction and labor augmentation.

Changes in fetal heart rate on cardiotocography and/or intrapartum meconium staining were considered changes in fetal well-being or the occurrence of acute fetal distress (AFD). Cesarean sections were indicated in those cases.

Three comparative-analysis models were created based on the type of delivery to analyze the studied variables and compare them with similar papers.

Analysis A: successful induction versus unsuccessful induction. Successful inductions (69 patients) included those that resulted in vaginal delivery and those that resulted in cesarean delivery because of cephalopelvic disproportion (labor evolved until incomplete expulsion). Unsuccessful inductions (11 patients) included cases that resulted in induction failure (that is, did not go into labor) and those that evolved to uncorrected functional dystocia (interrupted labor despite correction attempts). In this group, the patients who progressed to cesarean delivery because of AFD were excluded.

Analysis B: vaginal delivery versus cesarean delivery (excluding cases of AFD). In this group, patients who underwent vaginal delivery (62 patients) were compared with those who underwent cesarean section (18 patients) because of cephalopelvic disproportion, induction failure, or functional dystocia. As noted in analysis A, cesarean deliveries due to AFD were excluded from this group.

Analysis C: vaginal delivery versus cesarean delivery. Similar to analysis B, in this group we compared patients who underwent vaginal delivery (62 patients) with those who underwent cesarean delivery (28 patients); however, analysis C included cases of AFD.

The following patients were excluded from this study: those with missing data from their medical records (3 patients), and those undergoing cesarean delivery due to maternal death risk or family pressure (2 patients).

An additional analysis of a group including only the primiparous women from the three previous groups, and evaluating only BS ≤ 2 and cervical length ≥ 3.0 cm (that is, the cut-off points in this study), was conducted for comparison purposes.

Descriptive statistics was used to characterize the sample. The Fisher exact test or the Student t-test were used to assess the homogeneity of the variables where appropriate. For each variable studied, the validity of the diagnostic test was determined by measuring the sensitivity (SENS), the specificity (SPEC), and the positive predictive value (PPV) and negative predictive value (NPV). Odds ratios (ORs) and 95% confidence intervals (95%CIs) were calculated using the chi-squared test. The level of rejection of the null hypothesis was 5%. The GraphPad Prism (GraphPad Software, Inc., San Diego, CA, US) software, version 7.0, was used for every analysis.

## Results

The studied groups were homogeneous with regard to maternal age, ethnicity, and body mass index (BMI). Parity was directly correlated with a successful induction of vaginal delivery (Table 1). Two newborns presented with hypoxia and Apgar scores of 0/0/3 (rotation forceps and shoulder dystocia in a 3,978-g fetus) and 1/6/9 (natural birth of a 3,389-g fetus). All other patients presented with a 5-minute Apgar score > 8. Only one puerperal woman presented with a complication in childbirth (puerperal hemorrhage after vaginal delivery). She was treated with uterine curettage, and her condition resolved satisfactorily. In analysis A (successful versus unsuccessful labor induction; table 2), the variables that significantly increased the risk of failed vaginal delivery following induction were BS ≤ 2 and cervical lengths ≥ 2.0, 2.5, and 3.0 cm. Although not shown in Table 2, a cervical length ≥ 3.0 cm combined with additional ultrasound characteristics, and a BS ≤ 5, 4, 3, or 2 also increased the risk of failed labor induction.

**Table 2:** Analysis A: Unsuccessful labor induction (excluding AFD; n = 80)

Variable studied	TP	FP	FN	TN	SENS	(95%CI)	SPEC	(95%CIs)	PPV	(95%CI)	NPV	(95%CI)	OR	(95%CI)	*p*-value
*BS ≤ 5*	11	60	0	9	100.0	(71.5–100.0)	13.0	(6.1–23.3)	15.5	(14.3–16.7)	100.0	(-)	3.6	(0.2–66.5)	0.3876
*BS ≤ 4*	11	49	0	20	100.0	(71.5–100.0)	29.0	(18.7–41.2)	18.3	(16.2–20.7)	100.0	(-)	9.5	(0.5–169.3)	0.1248
*BS ≤ 3*	7	33	4	36	63.6	(30.8–89.1)	52.2	(39.8–64.3)	17.5	(11.3–26.1)	90.0	(19.9–95.3)	1.9	(0.5–7.1)	0.3356
*BS ≤ 2*	5	9	6	60	45.5	(16.7–76.6)	87.0	(76.7–93.9)	35.7	(18.6–57.4)	90.9	(85.2–94.5)	5.6	(1.4–22.0)	0.0147
*BS = 1*	1	1	10	68	9.1	(0.2–41.3)	98.6	(92.2–100.0)	50.0	(6.3–93.7)	87.2	(84.9–89.1)	6.8	(0.4–117.6)	0.1874
*Cervix ≥ 4.5 cm*	1	2	10	67	9.1	(0.2–41.3)	97.1	(89.9–99.6)	33.3	(4.7–83.5)	87.0	(84.7–89.0)	3.4	(2.8–40.4)	0.3414
*Cervix ≥ 4.0 cm*	1	4	10	65	9.1	(0.2–41.3)	94.2	(85.8–98.4)	20.0	(3.0–67.0)	86.7	(84.2–88.8)	1.6	(0.2–16.1)	0.6778
*Cervix ≥ 3.5 cm*	2	8	9	61	18.2	(2.3–51.8)	88.4	(78.4–94.9)	20.0	(5.7–50.7)	87.1	(83.5–90.0)	1.7	(0.3–9.3)	0.5432
*Cervix ≥ 3.0 cm*	9	13	2	56	81.8	(48.2–97.7)	81.2	(69.9–89.6)	40.9	(28.3–54.9)	96.6	(88.8–99.0)	19.4	(3.7–100.6)	0.0004
*Cervix ≥ 2.5 cm*	9	21	2	48	81.8	(48.2–97.7)	69.6	(57.3–80.1)	30.0	(21.4–40.3)	96.0	(87.2–98.8)	10.3	(2.0–51.8)	0.0047
*Cervix ≥ 2.0 cm*	10	33	1	36	90.9	(58.7–99.8)	52.2	(39.8–64.3)	23.3	(18.2–29.2)	97.3	(84.5–99.6)	10.9	(1.3–89.9)	0.0264
*Cervix ≥ 1.5 cm*	10	45	1	24	90.9	(58.7–99.8)	34.8	(23.7–47.2)	18.2	(14.7–22.2)	96.0	(78.2–99.4)	5.3	(0.6–44.2)	0.1207
*Cervix ≥ 1.0 cm*	11	59	0	10	100.0	(71.5–100.0)	14.5	(7.2–25.0)	15.7	(14.5–17.0)	100.0	(-)	4.1	(0.2–74.2)	0.3448
*Cervix ≥ 0.5 cm*	11	65	0	4	100.0	(71.5–100.0)	5.8	(1.6–14.2)	14.5	(13.8–15.2)	100.0	(-)	1.6	(0.1–31.4)	0.7641
*Absence of funneling*	10	47	1	22	90.9	(58.7–99.8)	31.9	(21.2–44.2)	17.5	(14.2–21.4)	95.7	(76.7–99.3)	4.7	(0.6–38.9)	0.1530
*Absence of internal os dilation*	9	44	2	25	81.8	(48.2–97.7)	36.2	(25.0–48.7)	17.0	(12.8–22.2)	92.6	(77.4–97.8)	2.6	(0.5–12.8)	0.2528
*Presence of CGA*	9	36	2	33	81.8	(48.2–97.7)	47.8	(35.6–60.2)	20.0	(14.9–23.3)	94.3	(82.1–98.3)	4.1	(0.8–20.5)	0.0832
*No changes when applying fundal pressure*	11	65	0	4	100.0	(71.5–100.0)	5.8	(1.6–14.2)	14.5	(13.8–15.2)	100.0	(-)	1.6	(0.1–31.4)	0.7641

Abbreviations: 95%CI, 95% confidence interval; AFD, acute fetal distress; BS, Bishop score; CGA, cervical gland area; FN, patients with false negative test results for the variable; FP, patients with false positive test results for the variable; NPV, negative predictive value (%); OR, odds ratio; PPV, positive predictive value (%); SENS, sensitivity (%); SPEC, specificity (%); TN, patients with negative test results for the variable; TP, patients with positive test results for the variable.

In analysis B (vaginal delivery versus cesarean delivery [excluding AFD]; Table 3), only BS ≤ 2 and cervical length ≥ 3.0 cm were significantly correlated with failed labor induction. As in analysis A, a cervical length ≥ 3.0 cm combined with additional ultrasound characteristics, and a BS ≤ 5, 4, 3, or 2 also increased the likelihood of cesarean section.

**Table 3:** Analysis B: Cesarean sections (excluding AFD; n = 80)

Variable studied	TP	FP	FN	TN	SENS	(95%CI)	SPEC	(95%CI)	PPV	(95%CI)	NPV	(95%CI)	OR	(95% CI)	*p*-value
*BS ≤ 5*	17	54	1	8	94.4	(72.7–99.9)	12.9	(5.7–23.8)	23.9	(21.4–26.7)	88.9	(51.7–98.4)	2.5	0.3–21.6	0.3996
*BS ≤ 4*	17	43	1	19	94.4	(72.7–99.9)	30.6	(19.6–43.6)	28.3	(24.5–32.6)	95.0	(73.2–99.2)	7.5	0.9–60.6	0.0584
*BS ≤ 3*	12	28	6	34	66.7	(41.0–86.7)	54.8	(41.7–67.5)	30.0	(21.9–39.6)	85.0	(73.9–91.9)	2.4	0.8–7.3	0.1140
*BS ≤ 2*	7	7	11	55	38.9	(17.3–64.2)	88.7	(78.1–95.3)	50.0	(28.8–71.2)	83.3	(77.4–88.0)	5.0	1.5–17.1	0.0104
*BS = 1*	2	0	16	62	11.1	(1.4–34.7)	100.0	(94.2–100.0)	100.0	(–)	79.5	(76.7–82.0)	18.9	0.9–413.9	0.0616
*Cervix ≥ 4.5 cm*	1	2	17	60	5.6	(0.1–27.3)	96.8	(88.8–99.6)	33.3	(4.6–83.9)	77.9	(75.8–79.9)	1.8	0.1–20.7	0.6509
*Cervix ≥ 4.0 cm*	2	3	16	59	11.1	(1.4–34.7)	95.2	(86.5–99.0)	40.0	(10.8–78.7)	78.7	(75.6–81.4)	2.5	0.4–16.0	0.3465
*Cervix ≥ 3.5 cm*	3	7	15	55	16.7	(3.6–41.4)	88.7	(78.1–95.3)	30.0	(11.0–59.8)	78.6	(74.5–82.1)	1.6	0.4–6.8	0.5462
*Cervix ≥ 3.0 cm*	10	12	8	50	55.6	(30.8–78.5)	80.6	(68.6–89.6)	45.5	(30.2–61.6)	86.2	(78.6–91.4)	5.2	1.7–16.0	0.0040
*Cervix ≥ 2.5 cm*	10	20	8	42	55.6	(30.8–78.5)	67.7	(54.7–79.1)	33.3	(22.4–46.4)	84.0	(72.3–90.2)	2.6	0.9–7.7	0.0775
*Cervix ≥ 2.0 cm*	13	30	5	32	72.2	(46.5–90.3)	51.6	(38.6–64.5)	30.2	(22.8–38.9)	86.5	(74.5–93.3)	2.8	0.9–8.7	0.0809
*Cervix ≥ 1.5 cm*	14	41	4	21	77.8	(52.4–93.6)	33.9	(22.3–47.0)	25.5	(20.1–31.6)	84.0	(67.4–93.0)	1.8	0.5–6.1	0.3521
*Cervix ≥ 1.0 cm*	17	53	1	9	94.4	(72.7–99.9)	14.5	(6.9–25.8)	24.3	(21.6–27.2)	90.0	(55.0–98.5)	2.9	0.3–24.4	0.3309
*Cervix ≥ 0.5 cm*	18	58	0	4	100.0	(81.5–100.0)	6.5	(1.8–15.7)	23.7	(22.5–24.9)	100.0	(-)	2.8	0.1–55.4	0.4898
*Absence of funneling*	16	41	2	21	88.9	(65.3–98.6)	33.9	(22.3–47.0)	28.1	(23.5–33.2)	91.3	(73.1–97.6)	4.1	0.9–19.5	0.0766
*Absence of internal os dilation*	15	38	3	24	83.3	(58.6–96.4)	38.7	(26.6–51.9)	28.3	(22.9–34.4)	88.9	(73.1–95.9)	3.2	0.8–12.0	0.0928
*Presence of CGA*	12	33	6	29	66.7	(41.0–86.7)	46.8	(34.0–59.8)	26.7	(19.6–35.2)	82.9	(70.5–90.7)	1.8	0.6–5.3	0.3148
*No changes when applying fundal pressure*	18	58	0	4	100.0	(81.5–100.0)	6.5	(1.79–15.7)	23.7	(22.5–24.9)	100.0	(-)	2.8	0.1–55.4	0.4898

Abbreviations: 95%CI, 95% confidence interval; AFD, acute fetal distress; BS, Bishop score; CGA, cervical gland area; FN, patients with false negative test results for the variable; FP, patients with false positive test results for the variable; NPV, negative predictive value (%); OR, odds ratio; PPV, positive predictive value (%); SENS, sensitivity (%); SPEC, specificity (%); TN, patients with negative test results for the variable; TP, patients with positive test results for the variable.

In analysis C (induction of vaginal delivery versus cesarean delivery [including AFD]; n = 90), the results were similar to those of analyses A and B. Bishop scores ≤ 4 (SENS = 92.9; SPEC = 30.6; PPV = 37.7; NPV = 90.5; OR = 5.7 [95%CI = 1.2-26.7]; *p* = 0.0257), 3 (SENS = 75.0; SPEC = 54.8; PPV = 42.9; NPV = 82.9; OR = 3.6 [95%CI = 1.4-9.8]; *p* = 0.0106), and 2 (SENS = 42.9; SPEC = 88.7; PPV = 63.2; NPV = 77.5; OR = 5.9 [95%CI = 2.0-17.5]; *p* = 0.0014), as well as cervical lengths ≥ 2.0 (SENS = 71.4; SPEC = 51.6; PPV = 40.0; NPV = 80.0; OR = 2.7 [95%CI = 1.0-7.0]; *p* = 0.0451) and 3.0 cm (SENS = 53.6; SPEC = 80.6; PPV = 55.6; NPV = 79.4; OR = 4.8 [95%CI = 1.8-12.7]; *p* = 0.016) were significantly correlated with induction failure. In addition, a cervical length ≥ 3.0 cm combined with other ultrasound characteristics and BS ≤ 5, 4, 3, or 2 increased the likelihood of cesarean section. The parity-related bias was eliminated by conducting an analysis exclusively using primiparous patients (Table 4). In this evaluation, only previously identified cut-off points were used. The risk of induction failure and the consequent need for cesarean section were increased in analyses B and C using a BS ≤ 2, as well as in analyses A, B, and C using a cervical length ≥ 3.0 cm. The combination of variables also increased the likelihood of induction failure and cesarean section.

**Table 4:** Analysis of only primiparous women

Analysis model	Variable studied	TP	FP	FN	TN	SENS	(95%CI)	SPEC	(95%CI)	PPV	(95%CI)	NPV	(95% CI)	OR	(95% CI)	*p*-value
**Analysis A: Labor induction**	*BS* ≤ *2*	5	7	6	34	45.5	(16.7-76.6)	82.9	(67.9-92.8)	41.7	(21.9-64.5)	85.0	(76.4-90.8)	4.0	(1.0-17.0)	0.0568
**Unsuccessful (excluding AFD) (**n = 52)	*Cervix* ≥ *3.0 cm*	9	5	2	36	81.8	(48.2-97.7)	87.8	(73.8-95.9)	64.3	43.1-81.1	94.7	(93.6-98.4)	32.4	(5.4-195.1)	0.0001
**Analysis B: Cesarean section**	*BS ≤ 2*	7	5	10	30	41.2	(18.4-67.1)	85.7	(69.7-95.2)	58.3	(34.2-79.0)	75.0	(66.3-82.0)	4.2	(1.1-16.2)	0.0376
**(excluding AFD)** (n = 52)	*Cervix ≥ 3.0 cm*	10	4	7	31	58.8	(32.9-81.6)	88.6	(73.3-96.8)	71.4	(47.8-87.2)	81.6	(71.2-88.8)	11.1	(2.7-45.8)	0.0009
**Analysis C: Cesarean section**	*BS ≤ 2*	12	5	15	30	44.4	(25.5-64.7)	85.7	(69.7-95.2)	70.6	(49.0-85.7)	66.7	(58.2-74.2)	4.8	(1.4-16.2)	0.0113
**(including AFD)** (n = 62)	*Cervix ≥ 3.0 cm*	15	4	12	31	55.6	(35.3-74.5)	88.6	(73.3-96.8)	78.9	(58.4-90.9)	72.1	62.5-80.0)	9.7	(2.7-35.1)	0.0006

Abbreviations: 95%CI, 95% confidence interval; AFD, acute fetal distress; BS, Bishop score; FN, patients with false negative test results for the variable; FP, patients with false positive test results for the variable; NPV, negative predictive value (%); OR, odds ratio; PPV, positive predictive value (%); SENS, sensitivity (%); SPEC, specificity (%); TN, patients with negative test results for the variable; TP, patients with positive test results for the variable.

## Discussion

In every analysis, the isolated variables (BS ≤ 2 and cervical length ≥ 3.0 cm) yielded significant results. A BS ≤ 2 yielded satisfactory SPEC and NPV in analyses A, B, and C. This finding is similar to the results obtained in other studies that used different BSs and methods of evaluating successful labor induction, including the systematic reviews by Hatfield et al (2007)^25^ (which included 3 studies with a BS of 5; 5 studies with a BS of 4; and 2 studies with a BS of 3), Papillon-Smith and Abenhaim (2015)^26^ (which included 2 studies with a BS of 2), and Ezebialu et al (2015)^24^ (which did not find that the BS was statistically valid).

A cervical length ≥ 3.0 cm exhibited satisfactory SPEC and NPV in analyses A, B, and C, as well as adequate SENS in analysis A. These findings are similar to those of many other studies (numerous studies found a similar result for the BS) that used different BSs for cervical size and methods of evaluating labor induction success. Papillon-Smith and Abenhaim (2015)^26^ included 1 study with a cervical length cut-off point of 3.4 cm; 6 studies with a cut-off point of 3.0 cm; 2 studies with a cut-off point of 2.8 cm; 1 study with a cut-off point of 2.7 cm; 5 studies with cut-off points between 2.4 and 2.6 cm; and 5 studies with cut-off points < 2.4 cm. We used 3.0 cm as a cut-off point to improve the diagnostic test values of the variables examined by combining them with every morphological cervical change (that is, funneling, internal os dilation, CGA, and the morphological changes in the cervix as a result of applying fundal pressure) and each other. The combination of variables resulted in partial improvement, but it was not sufficient to justify its clinical use. The isolated analysis of these variables was simpler and sufficient, and other studies cited in systematic reviews such as those by Hatfield et al (2007)^25^ and Papillon-Smith and Abenhaim (2015)^26^ corroborate this result.

The group from analysis A (successful versus unsuccessful labor induction) was created to evaluate unsuccessful induction. Therefore, cases of cesarean delivery due to cephalopelvic disproportion (diagnosed in our study during the expulsion period) were considered cases of successful induction because they did not exclusively result in vaginal delivery, given the bone incompatibility that was not previously detected by the obstetrician. In contrast, the group with unsuccessful labor induction included cases of induction failure (the absence of response to the induction protocol) and functional dystocia; the latter was due to labor that did not evolve satisfactorily despite corrective attempts, thereby suggesting that labor induction was not effective. Nevertheless, two studies considered significant by Ezebialu et al (2015)^24^ evaluated cases involving changes in fetal well-being; however, these cases were removed from our analysis because common sense suggests that the cervix is not involved in changes in fetal well-being, nor do these studies suggest a significant difference among the studied groups with regard to fetal well-being.

In the group from analysis B (vaginal delivery versus cesarean delivery, excluding AFD), the cases undergoing cesarean delivery due to cephalopelvic disproportion were combined with cases that resulted in cesarean delivery due to induction failure or functional dystocia. Cases involving AFD were removed from the analysis for the same reason as noted in analysis A.

The group from analysis C (vaginal delivery versus cesarean delivery) was similar to the group from analysis B, except that indications for cesarean section due to AFD were included. This group best reflects the profile of almost every other study compiled in the systematic reviews on this topic.^24^ ^25^ ^26^ ^27^

In the present study (Table 1), only 1 case (a non-primiparous woman with a previous natural birth) out of 28 evolved into cesarean delivery. This procedure was indicated due to cephalopelvic disproportion, which we regard as successful induction (birthweigth: 3,626 g). This result is an unequivocal demonstration that parity is directly correlated with the success of labor induction.

The analyses of only the primiparous women in groups A, B, and C, BS ≤ 2, and cervical length ≥ 3.0 cm (Table 4) indicated improvement in the quality of the diagnostic tests compared to the previous analysis that included several parity groups, particularly with regard to PPV. The PPV might be close to 80% in the group from analysis C when the cervical length is ≥ 3.0 cm. Therefore, for every 5 primiparous patients undergoing a cesarean delivery due to cervical length ≥ 3.0 cm, regardless of the indication, only 1 patient progressed to vaginal delivery using our labor induction protocol.

A major difficulty encountered by systematic reviews is the great heterogeneity of the population characteristics, such as the presence or even the mention of maternal and/or fetal comorbidities, the criteria to consider an induction successful or unsuccessful, and the GA at which TVS analyses are performed or when labor induction is initiated, in addition to many other minor aspects.^24^ ^25^ ^26^ ^27^ In the present study, we attempted to emulate most of the previous work as much as possible; we created the analysis C group (vaginal delivery versus cesarean delivery, regardless of duration), in which only 11 out of the 20 studies analyzed by Hatfield et al (2007)^25^ fit. The others argue that delivery should occur < 24 hours after the onset of labor or simply enter the active phase of labor, which is a more heterogeneous and difficult issue to distinguish according to Papillon-Smith and Abenhaim (2015)^26^ and Verhoeven et al (2013).^27^ We evaluated the cervical size in conjunction with the BS. A total of 10 out of 20 papers performed this analysis and obtained similar results to ours, according to Hatfield et al (2007)^25^; 11 out of 31 papers obtained similar results according to Papillon-Smith and Abenhaim (2015).^26^ We evaluated other characteristics of the cervix, but in contrast to other systematic reviews, we did not find significance; in fact, only 5 out of 20 studies evaluated other cervical characteristics and found significance, according to Hatfield et al (2007)^25^; 8 out of 31 studies evaluated other cervical characteristics, and half of them found significance according to Papillon-Smith and Abenhaim (2015)^26^; and 8 out of 31 studies evaluated other cervical characteristics and did not find significance in the meta-analysis according to Verhoeven et al (2013).^27^ We created a unique analysis of primiparous women (Table 4) to prevent any parity-related bias. A limited number of studies also performed this analysis according to Hatfield et al (2007),^25^ and only 5 out of 31 found results identical to ours according to Verhoeven et al (2013).^27^

None of the variables evaluated in these groups should be used to indicate cesarean delivery without the need for attempted induction, which is consistent with the extant systematic reviews on the subject.^24^ ^25^ ^26^ ^27^ The use of these variables as indications for cesarean section is possible in cases in which the adopted test presents with satisfactory SENS and SPEC values and the PPV is close to 100%. Importantly, this study observed this characteristic in analyses B and C with a BS of 1, although the significance of these variables was poor (*p* > 0.05), and the SENS was low (close to 10%).

## Conclusion

Transvaginal sonography before the onset of labor in patients > 40 weeks of gestation is a useful tool to predict failed labor induction or cesarean delivery. However, TVS should not be used to justify cesarean delivery using the argument that labor induction might fail in high-risk cases. More studies with larger sample sizes are necessary to address this question more conclusively.
